# Association between poor clinical prognosis and sleep duration among breast cancer patients**^1^**

**DOI:** 10.1590/1518-8345.1826.2899

**Published:** 2017-06-05

**Authors:** Thalyta Cristina Mansano-Schlosser, Maria Filomena Ceolim

**Affiliations:** 2 PhD.; 3 PhD, Associate Professor, Faculdade de Enfermagem, Universidade Estadual de Campinas, Campinas, SP, Brazil.

**Keywords:** Sleep, Breast Neoplasms, Depression, Nursing, Hope.

## Abstract

**Objective:**

to investigate the association between clinical progression and the quality and duration of sleep in women with breast cancer.

**Method:**

longitudinal study, with 114 participants, conducted in a hospital in Brazil. The instruments used were: questionnaire for sociodemographic and clinical characterization, Pittsburgh Sleep Quality Index; Beck Depression Inventory and Herth Hope Scale. Data were analyzed through descriptive statistics and survival analyses (outcome: poor clinical progression), using the Kaplan-Meier curve, Log-rank test and Cox proportional model.

**Results:**

a higher probability of poor clinical progression was verified in women with sleep durations of less than six hours or nine hours and over (p=.0173).

**Conclusion:**

the results suggest the importance of further studies that seek to verify whether the quantitative management of sleep disorders would have an impact on the progression of breast cancer. Women should be encouraged to report sleep problems to nurses.

## Introduction

The clinical outcome of breast cancer and the survival of the women affected depend on some extensively studied prognostic factors, such as: the presence of lymph node metastases, the size and histological type of the tumor, its nuclear grade, the estrogen (ER) and progesterone (PR) hormone receptor status, the presence of the Human Epidermal growth factor Receptor 2 (HER-2) oncogene and the tumor cell proliferation^1^.

The probability of survival for five years for patients with early stage I cancer was estimated to be 97%, while for women with the more advanced stage (stage IV) this was 57%, according to a study of 252 women with breast cancer at a university hospital^2^. It also demonstrated that the probabilities of survival for ten years for the initial and advanced stage, were 97% and 0%, respectively^2^.

Recently, the literature has shown an association between the prognosis and clinical progression of breast cancer and sleep of poor quality or of insufficient or excessive amounts for the needs of the individual^3^. A study that aimed to verify an association between breast cancer symptoms and poor quality of sleep concluded that 65% of the women suffered from sleep problems^4^. This study is relevant considering that the literature suggests that poor sleep quality, in addition to having a negative impact on the daily routine of the affected patient, may be associated with poor clinical evolution of the cancer. The sleep characteristics that contribute to poor quality include; fragmented sleep and, consequently, its low efficiency; prolonged latency and early awakening; and a non-restorative sensation of sleep^3^; Thus, the set of results can be interpreted and referred to as poor quality sleep.

In a recent study, in 2016, with 1011 people, between 35 and 45% presented problems of either duration or quality of the sleep and the authors concluded that in Australia sleep problems are an endemic health problem that needs interventions at policy level^4^. Thus, it was hypothesized that the poor quality of sleep, as well as inadequate duration, could constitute aggravating factors in the clinical progression of women with breast cancer.

According to population and evidence based studies found in the literature, the excessive or insufficient duration of sleep may be related to an increased risk for breast cancer, which highlights the requirement for more studies on this topic^5^ ^-^ ^6^. Sleep restriction produces a stress reaction in the body, being best characterized immunologically as an increase in the leukocyte and neutrophil count and C-reactive protein serum levels. Authors have shown that a single night of sleep restriction may be sufficient to cause this increase and that to return to the base count requires an recovery time of eight to ten hours of sleep^7^.

Stress has been associated with the progression of cancer, particularly breast cancer^8^. It should be highlighted that stress increases the synthesis of glucocorticoids, which alters the immune response, as well as cell proliferation and apoptosis in various tissues, and may be one of the mechanisms by which sleep deprivation is associated with increased incidence of breast cancer^9^.

Mutilating treatment, which is often necessary, can cause women to undergo alterations in their self-image, functional loss and mental, emotional and social changes, frequently leading to depression^10^. Authors have verified that poor quality of sleep in patients with cancer is associated with the presence of depression, anxiety, pain and impairment of the sense of well-being, factors that also affect the quality of life of people with cancer^11^. In contrast, in several studies, it is suggested that hope can be an effective strategy to help patients cope with the difficulties and achieve their goals, especially regarding cancer patients^11^.

Globally, breast cancer is the second most common type of cancer and the most common in women. It is the leading cause of cancer death in women worldwide, with an estimated 520,000 deaths in 2012^12^. It can be considered an epidemic and, in Brazil, its high incidence and mortality due to late diagnosis has been verified^12^. These data demonstrate the relevance of breast cancer related studies. 

Thus, the aim of this study was to investigate the association between clinical progression and quality and duration of sleep in women with breast cancer.

## Methods

Design: a longitudinal and analytical study, with a mean follow-up period of 15 months (*SD*=4.2), ranging from one to 21 months. The main outcome was that the women with sleep duration of less than six hours or nine hours and over presented greater probability of poor clinical progression at the end of the follow-up period when compared to the women with between six and nine hours sleep duration. 

Location: Surgical Oncology Ward and Breast Cancer Clinics of a university hospital specialized in women's health in the state of São Paulo, Brazil. 

Population and sample: a convenience sample of 156 women was selected from the women recently diagnosed with breast cancer in the service mentioned; due to data not recovered from the medical records, 114 women were included in the study. All the women who met the inclusion criteria were included in this study. The following criteria were used for the selection: Inclusion criteria: women 18 years of age or over; diagnosed with breast cancer, T_qq_N_qq_M_0_ of any stage (T_qq_= any tumor extent; N_qq_= any regional lymph node invasion, M_0_= clinically no distant metastasis)^13^; who agreed to participate in the study. Exclusion criteria: Karnofsky Scale score lower than 70 (capable of caring for oneself, incapable of normal activity or work); inadequate medical (such as mucositis, pain, nausea, shortness of breath, vomiting) or emotional (such as crying, apathy, aggressiveness) conditions to respond to an interview. The women who left the study for reasons unrelated to the expected outcome were considered dropouts.

Data collection: the researcher collected the data, with the patients being recruited prospectively. First contact was made when the woman, previously diagnosed with breast cancer, was hospitalized for surgery. All instruments except the Sociodemographic Questionnaire were used at each encounter with the women. Clinical data were also collected at the end of the study for comparison and analysis of the disease progression. The endpoint of the study was poor clinical progression, defined as the appearance of new lymph node metastases or distant metastases, with the data collected from the medical records when the patient returned for clinical treatment (chemotherapy or radiation), with a mean follow-up period of 15 months.

Regarding the prognostic factors for control, the following were considered: the presence of lymph node metastases, the stage, I, II or III, and size of the tumor, the estrogen (ER) and progesterone (PR) hormone receptor status, and the presence of the Human Epidermal growth factor Receptor 2 (HER-2) oncogene^2^.

Instruments used: 

Sociodemographic and Clinical Characterization Questionnaire - evaluated by a committee of judges, specialists in oncology and sleep, applied at the beginning of the study for the sociodemographic and clinical characterization of the participants. The clinical data were obtained from the medical records. 

Pittsburgh Sleep Quality Index - PSQI-BR, validated in Brazil^14^. The scale subjectively assesses sleep quality and disturbances for the previous month. It contains 19 questions grouped into seven components: subjective sleep quality, latency, duration, efficiency, sleep disturbances, use of sleep medication and daytime dysfunction. The total score ranges from 0-21 points, with scores higher than five indicating poor quality of sleep. The higher the value obtained, the worse the evaluation of sleep quality, and the overall score of five points constitutes the cut-off point that distinguishes between subjects with poor sleep and sleep disorders (above five points) and those with good quality sleep (five points or less). The full PSQI was used for investigation of the association with the overall score.

Beck Depression Inventory - BDI, validated in Brazil^15^. The original scale consists of 21 items, including symptoms and attitudes, with their intensity varying from zero to three. The items refer to sadness, pessimism, sense of failure, lack of satisfaction, guilt, feeling of punishment, self-deprecation, self-accusations, suicidal ideation, crying spells, irritability, social withdrawal, indecisiveness, distortion of body image, inhibition to work, sleep disorders, fatigue, loss of appetite, weight loss, somatic concern, and decreased libido. The recommended cutoff points are: less than ten points, no or minimal depression; 10 to 18, slight to moderate depression; 19 to 29, moderate to severe depression; 30 to 63 points, severe depression. In this study, subjects in the first category were grouped as 'without depression' and those in the other three as 'with depression'.

The Herth Hope Scale - HHS is a self-report scale validated for use in Brazil, with adequate psychometric properties^16^. It is designed to facilitate the assessment of hope at various intervals from which variations in its levels can be identified. The instrument consists of 12 statements with answers given on a Likert type scale in which the scores range from one to four, with the following answer possibilities: strongly disagree, disagree, agree and strongly agree. The total score ranges from 12 to 48 points, with higher scores indicating higher levels of hope. There is no cutoff point on this scale. 

Data analysis: performed using the SAS 9.4 program with help from a statistician, including descriptive statistics and survival analysis. Kaplan-Meier curves were used to visualize the outcome occurrence pattern, defined as poor clinical progression, for the entire sample and for the following groups: good and poor sleep quality; sleep duration of less than six or more than nine hours, and sleep duration from six to nine hours, presence or absence of depression; presence or absence of each prognostic factor, separately; Log-rank test to assess whether the differences found in the outcome occurrence, between the curves of different groups, presented statistical significance; and Cox proportional hazards model, to adjust for the effect of the covariates. The HHS score (Hope) and tumor size, as numeric variables, could not be included in the log-rank test and were therefore analyzed using the Mann-Whitney test, as a function of poor clinical progression. The *p* value <.05 was adopted as the critical level for all the tests. Reliability of the PSQI-BR was assessed using Cronbach's alpha coefficient, with a value of 0.721.

Each woman gave their written informed consent at the first data collection moment. Ethical considerations: the study was approved by the Research Ethics Committee of the affiliated institution of the authors under authorization number 44169 (CAAE 00762112.0.0000.5404) and its amendment approved under authorization No. 1.106.951, fulfilling all the legal requirements for studies with human subjects according to regulation 466/2012.

## Results

The population was characterized as having a mean age of 55.9 years (*SD*=11.7, median=47.0) and mean education of 5.6 years (*SD*=4.1, median=3.0). 

The majority of the participants were white (72.8%), married (59.7%) lived with family members (86.8%) and received incomes of up to five minimum salaries (88.6%). A total of 46.5% reported being retired or on sick leave from work. Regarding the clinical data, the majority reported having no other chronic disease (59.7%), not smoking (83.3%) and using medications at home (61.4%). A significant proportion reported experiencing symptoms related to menopause (41.2%) and feeling pain related to the tumor (38.1%). The majority of the women had not performed neoadjuvant chemotherapy (73.2%), had the tumor in their right breast (51.8%), and wrote with the same hand (94.4%); regarding the surgery performed, 57.9% underwent full mastectomy.

The prognostic factors were distributed as follows: the majority of the tumors were in stage I or II (71.9%); with a mean size of 3.0cm (*SD*=2.04, median=2.5); 64.8% presented Nottingham histological grade 2 and 66.7% had lymphatic invasion; 78.9% were positive for estrogen receptors, 72.8% for progesterone receptors, and 50% of the cases positive for HER2. 

Sleep duration showed a mean of 6.5 hours (*SD*=1.9, median=7.0), with less than six hours or nine hours and more reported by 38 of the women (33.3%). The sleep quality, according to the PSQI-BR, was poor for 64 of the women (56.1%). Depression, according to the BDI, was present for 62 of the women (54.4%) and the mean score of the HHS was 34.6 (*SD*=6.4, median=35).

The number of women that presented poor clinical progression during the follow-up was 17 (14.9%). The distribution of the patients according to quality and duration of sleep, presence of depression and prognostic factors, as a function of the clinical progression, is presented in Table 1.

**Table 1:** Distribution of the women with breast cancer according to quality and duration of sleep, presence of depression and prognostic factors, as a function of the clinical progression. (n=114). Campinas, SP, Brazil, 2013-2014

Variable		Poor clinical progression				
		No			Yes	
		N	%		N	%
Sleep quality						
	Good quality	43	86.0		07	14.0
	Bad quality	54	84.4		10	15.6
Sleep duration						
	<6 or >9 hours	28	73.7		10	26.3
	>6 and <9 hours	69	90.8		07	9.2
Depression						
	Without depression	47	90.4		05	9.6
	With depression	50	80.7		12	19.4
Stage*						
	I	22	84.6		04	15.4
	II	49	87.5		07	12.5
	III	26	81.3		06	18.8
HER2**^†^** status						
	Negative	49	86.0		08	14.0
	Positive	48	84.2		09	15.8
Estrogen receptor						
	Negative	19	79.2		05	20.8
	Positive	78	86.7		12	13.3
Progesterone Receptor						
	Negative	26	83.9		05	16,1
	Positive	71	85.5		12	14.5
Lymphatic invasion						
	Negative	33	86.8		05	13.2
	Positive	64	84.2		12	15.8

*According to the UICC TNM Classification, 2004

†Human Epidermal growth factor Receptor-type 2

Table 2 shows the values of the log-rank test for the probability curves of absence of poor clinical progression, according to the following variables: sleep quality and duration, presence or absence of depression and prognostic factors. Only sleep duration presented a significant difference.

**Table 2:** Log-rank test for the probability curves for absence of poor clinical progression as a function of the variables: sleep quality, sleep duration, depression and prognostic factors, in women with breast cancer (n=114) Campinas, SP, Brazil, 2013-2014.

Variable	***P*** -value*
Sleep Quality	.8015
Sleep Duration	.0173
Depression	.1378
Tumor Stage	.6787
Lymphatic invasion	.7201
Estrogen receptor	.3497
Progesterone receptor	.8373
HER2**^†^** status	.7816

*Log Rank Test

†HER2: Human Epidermal growth factor Receptor-type 2

**Figure 1:**
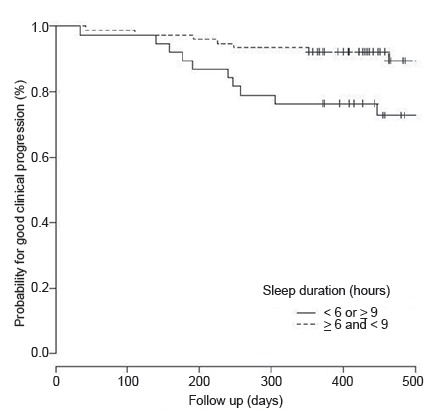
Probability curve for absence of poor clinical progression at the end of the follow-up, according to the duration of sleep in women with breast cancer. (n=114). Campinas, SP, Brazil, 2013-2014.

In Figure 1, the probability curves for absence of poor clinical progression, at the end of the follow-up, are shown for the women, grouped according to sleep duration, the variable for which the difference was significant. It was observed that the probability was less than 75% for those with sleep durations of less than six hours or nine hours and over, while approximately 90% for those with duration between six and nine hours. 

*p-value: Log Rank Test

Table 3 shows that no factors were independently associated with poor clinical progression, according to the Cox proportional hazards models.

**Table 3:** Association between factors and the probability of poor clinical progression, at the end of the follow-up, in women with breast cancer (n=114). Campinas, SP, Brasil, 2013-2014

Independent variables		Risk ratio*	Confidence interval		***P*** -value
			L. L.	U. L.	
Model 1					
	Sleep quality (ref*: good)	0.78	0.27	2.29	.6538
	Presence of depression (ref: absence)	1.56	0.46	5.32	.4818
	Stage III (ref: stage I)	0.79	0.15	4.07	.7759
	Stage II (ref: stage I)	0.70	0.20	2.45	.5753
	HER2**^†^** status (ref: negative)	1.00	0.37	2.70	.9947
	Estrogen receptor (ref: negative)	1.54	0.37	6.44	.5515
	Progesterone receptor (ref: negative)	0.73	0.20	2.73	.6442
	Lymphatic invasion (ref: negative)	0.89	0.29	2.79	.8459
	HHS**^‡^** score	0.94	0.86	1.02	.1396
	Tumor size	1.08	0.86	1.35	.5352
Model 2					
	Sleep duration (ref : >6 and <9)	2.73	0.99	7.52	.0518
	Presence of depression (ref: absence)	1.22	0.35	4.33	.7565
	Stage III (ref: stage I)	0.76	0.15	3.90	.7438
	Stage II (ref: stage I)	0.73	0.21	2.56	.6169
	HER2**^†^** status (ref: negative)	1.08	0.39	3.00	.8768
	Estrogen receptor (ref: negative)	1.64	0.37	7.16	.5138
	Progesterone receptor (ref: negative)	0.80	0.20	3.20	.7502
	Lymphatic invasion (ref: negative)	0.70	0.22	2.20	.5422
	HHS**^‡^** score	0.94	0.86	1.03	.2093
	Tumor size	1.06	0.84	1.33	.6444

*Ref: reference category

†HER2: Human Epidermal growth factor Receptor-type 2

‡HHS: Herth Hope Scale.

A comparison of the HHS scores showed no significant difference (*p*=.0547) between women with poor clinical progression (31.5 points, *SD*=8.4, median=30.0) and unchanged progression (mean 35.1 points, *SD*=5.9, median=35.0). Similarly, there was no significant difference (*p*=.2563) for tumor size, between the groups with poor progression (3.46cm, *SD=*2.14, median=3.0) and unchanged progression (2.92cm, *SD*=2.02, median=2.5).

## Discussion

The probability of presenting poor clinical progression at the end of the follow-up period was greater for the women with sleep duration of less than six hours or nine hours and over. The other variables analyzed, including sleep quality, showed no influence on the outcome. Furthermore, no variables had an independent effect on the probability of the occurrence of the outcome, when analyzed together. 

In the literature, there are few studies on the consequences of poor quality or inadequate duration of sleep for the health of people with breast cancer. The majority of these studies refer to cardiovascular diseases and metabolic syndrome^17^. There are a more significant number of studies that attempt to associate the quantitative and qualitative changes of sleep with the risk of developing breast cancer, however, they do not investigate the possible consequences of sleep disorders on the progression of the disease^2^. Thus, there is a gap, which this study sought to address.

In a recent consensus of the American Academy of Sleep Medicine, specialists highlighted that sleep duration of less than seven hours is associated with, among other health problems, an increased risk of mortality and damage to the immune system^17^. However, according to the authors, there is insufficient evidence to show that sleeping longer than nine hours is associated with health problems^17^.

Sleep duration is the most frequently investigated sleep measure in relation to health. Sleep is essential for optimal health and experts used a total of 5,314 scientific articles to compose a panel about sleep. The panel focused on nine health categories with the best available evidence in relation to sleep duration: general health, cardiovascular health, metabolic health, mental health, immunologic function, human performance, cancer, pain, and mortality^17^. The adverse outcomes related to insufficient sleep are stress, pain, neurocognitive dysfunction, psychiatric symptoms, and mortality^18^. Specialists have also emphasized the importance of the subjective evaluation of this parameter, as well as the relevance of differences among individuals, stating that people who realize that they are sleeping very little, or too much, should be instructed to seek a health professional^19^_._

It was possible to show that people who had grade-3 tumors, or the fastest growing, actually had fewer hours of sleep compared to others. New studies linking sleep apnea and other disruptions to cancer add to evidence that poor sleep can be deadly^20^. 

The majority of the women in this study (56.1%) presented poor quality sleep, a slightly lower percentage to that found in another study, in which 61% of the women with breast cancer presented poor quality sleep at the start of the treatment^21^. Regarding the mean sleep duration of 6.5 hours found in the present study, this was lower than that found by other authors, of 7.0 hours at the start of the treatment in women with breast cancer^22^.

The majority of the women (54.4%) of this study presented depression, according to the BDI. This corroborates another study, in which 56% of the women with breast cancer presented depressive symptoms, indicating that the management of these symptoms in these women is necessary and should be a priority^23^. The literature shows that breast cancer is commonly associated with various symptoms, such as depression, pain, fatigue and poor sleep quality^9^ ^,^ ^24^.

 In this study, depression, although present in the majority, presented no influence on the clinical progression of the disease. A recent meta-analysis demonstrated the efficacy of interventions for psychosocial outcomes following breast cancer surgery, with cognitive behavioral therapy promoting improvements in anxiety, depression, and quality of life^24^.

The hope scores, for the women with poor clinical progression, were slightly above the mean of the possible variation, showing that studies directed toward the development of strategies are necessary to encourage hope in these women. Hope is an underinvestigated variable and has the potential to become an effective tool for use in the quotidian of health professionals^25^, however, in this study, it presented no effect on the disease progression. 

Similarly, there was no significant relationship between the prognostic factors of the cancer and clinical progression by the end of the follow-up period. Other authors, in analysis of estimated survival over five years, found the following to be factors associated with the risk of death: tumor size, lymph node involvement, number of lymph nodes removed and estrogen and HER-2 receptor tumor markers^2^. This was, however, a retrospective study, using a longer follow-up period than the present study, as is the case for the majority of the studies that take this approach, which are generally cohort studies with a five to ten year follow-up period^2^.

Some limitations of the study can be highlighted, including the length of the follow-up period, this being short for the expected outcome, and the incompleteness of the data obtained from the medical records. As implications for the practice, this study indicates the need for a detailed assessment of the duration of the sleep of women with breast cancer, as well as for guidance related to reporting dissatisfaction with sleep to the health professional, whether due to lack, excess or poor quality. Women should be encouraged to make this report spontaneously, as many professionals do not address this issue, considering that sleep may represent a modifiable risk factor for disease progression.

In addition, women need to be encourage to talk about other symptoms, such as depression, that have to be treated throughout the treatment because this can influence the quality of sleep and quality of life. To encourage hope in these women is also necessary, as this can help patients who live with cancer. Therefore, these variables were addressed with the nursing teams of the service where the research was carried out, aiming to incorporate them into the clinical practice throughout the treatment. The professionals began to ask about the sleep quality and depressive symptoms, with this study presenting the novelty of strengthening hope in these patients.

The study, although longitudinal, did not aim to establish causal relationships between the variables. However, the results obtained emphasize the importance of further studies that seek to verify whether the quantitative management of sleep disorders, i.e. duration insufficient or excessive for individual needs, would have an impact on the progression of breast cancer. Furthermore, it is suggested that future studies consider a longer follow-up period, complemented with objective measurements that allow a longitudinal and prospective evaluation of the wake/sleep cycle, for example, the use of actigraphy and a sleep diary, with the prospective collection of clinical follow-up data.

## Conclusion

The women with sleep duration of less than six hours or nine hours and over presented greater probability of poor clinical progression at the end of the follow-up period when compared to the women with sleep duration between six and nine hours. There was no influence for any of the other variables on the progression of the disease. When analyzed together with the other variables, sleep duration was not maintained as an independent risk factor for the probability of poor clinical progression. 
